# Functional limitation in the older Brazilian adults: Association with multimorbidity and socioeconomic conditions

**DOI:** 10.1371/journal.pone.0294935

**Published:** 2023-11-30

**Authors:** Marina Mendes Lopes Vieira, Viviane Santos Borges, Eduardo José Pereira Oliveira, Fabíola Bof de Andrade

**Affiliations:** 1 René Rachou Institute, Oswaldo Cruz Foundation (FIOCRUZ), Belo Horizonte, Minas Gerais, Brasil; 2 Universidade de Itaúna, Itaúna, Minas Gerais, Brasil; 3 Secretary of Health of the State of Minas Gerais, Minas Gerais, Brasil; Polytechnic Institute of Viana do Castelo, PORTUGAL

## Abstract

The aim of this study was to assess the association between multimorbidity and the presence of functional limitation in basic (BADL) and instrumental activities of daily living (IADL) among Brazilian older adults and to verify whether this association is moderated by socioeconomic conditions. Cross-sectional study with data from the Brazilian National Health Survey (PNS) (2019) for the Brazilian population aged 60 years and over. The dependent variables were functional limitation, based on self-reported difficulty in performing one or more activities of daily living, including six BADL (feeding, bathing, using the toilet, dressing, crossing a room on the same floor and getting out of bed) and four IADL (shopping, managing money, taking medication and using transportation). The independent variables were multimorbidity (presence of two or more self-reported chronic diseases) and socioeconomic measures (per capita household income, asset score, and education level). The association between multimorbidity and outcomes was assessed using adjusted logistic regression models. The moderating effect of socioeconomic conditions on the association between multimorbidity and functional limitations was assessed by including an interaction term. The final sample consisted of 22,725 individuals. The prevalence of functional limitation was 8.5% (95%CI: 7.9–9.2) and 18.6% (95%CI: 17.8–19.5) in BADL and IADL, respectively. Multimorbidity was associated with BADL [OR: 2.30 (95%CI: 1.93–2.74)] and IADL [OR: 2.26 (95%CI: 1.98–2.57)]. The odds of functional limitation were higher among individuals with lower levels of education and income, but there was no interaction between multimorbidity and socioeconomic position measures. Multimorbidity was associated with functional limitation (BADL and IADL) and socioeconomic conditions, and this association was constant across socioeconomic position levels.

## Introduction

Functional limitations may be the early stages of disability among older adults [1], being defined as the need to receive assistance to perform activities of daily living (ADL), including basic, instrumental and advanced activities [2, 3]. The greater the support required and the more basic is activity with limitations, the greater the dependence [4]. Worldwide approximately 142 million people aged 60 years and older have limitations for activities of daily living (i.e., dressing, taking medication and managing money) [5]. These limitations have a negative impact on people’s ability to perform independently their activities in the community and contribute to increased care needs [6, 7]. Thus, these limitations directly affect an individual’s functional ability which is an essential element for healthy aging [5].

For the next three decades, it is estimated that the population of older adults will exceed 1.5 billion people and this increase will occur especially in developing countries [8]. In Brazil, in 2060, the number of people aged 60 years or older is estimated to exceed the 70 million mark, representing more than 30% of the country’s population [9]. Associated with the demographic transition experienced in Brazil, the change in the epidemiological profile has been characterized by an increase in non-communicable diseases (NCDs). These represent the main burden of diseases and deaths in the population, constituting an important public health problem, whose proportion increases with age, reaching almost 90% among individuals aged 70 years or more [10]. In 2008, 63% of the world’s 36 million deaths were due to NCDs, with older adults and those with low socioeconomic position being the most affected [11].

It is estimated that 15% of the world’s population has one or more NCDs that lead to disability and, among these individuals, more than 46% are older adults who already have some degree of functional limitation. Global trends show that, in parallel to the number of older people, the number of people with disabilities will also increase [12]. This scenario brings significant economic and social challenges for health and social care systems worldwide, as both NCDs [13] and functional limitations are associated with increased needs for highly complex treatments, hospital admissions and demands for costly technologies [14].

According to the baseline report of the Decade of Healthy Ageing [5], 14% of the population have difficulties performing some activity of daily living (e.g. ability to dress; ability to take medication; and ability to manage money). In all countries, older people with higher levels of education had fewer limitations compared to those with primary education or no formal education. When it comes to attenuating the impact of functional limitation in the older population, it is of the greatest importance to consider socioeconomic factors, especially investment in education, as the latter, in many countries, contributes much more to functional limitation than the gender gap itself [5].

In addition to the influence of schooling, some findings show that individuals with lower income [15, 16] or who live in areas of lower socioeconomic status [15, 17–20] have a higher prevalence and incidence chronic conditions [21, 22] and functional limitation [15, 23–25].

The impact of individual chronic conditions on functional limitations among older adults has been explored in the literature [26, 27]. Despite this, the association of functional limitations with the presence of multimorbidity is still a growing field [28, 29]. This last defined as the presence of two or more NCDs [14], affects approximately 24.2% of the Brazilian population aged 18 years and over and 67.8% of individuals aged 50 years and over [30].

Previous cross-sectional studies have shown that a significant proportion of people with multimorbidity have some type of limitation in ADL [31, 32]. Findings from longitudinal studies are mixed according to the functional limitations investigated [33, 34]. In Brazil, one study suggested that the prevalence of functional limitations was higher among individuals with different multimorbidity profiles [32]. In contrast, a longitudinal study in Norway found no association between complex multimorbidity (3 or more chronic diseases) and functional limitations in basic activities of daily living [35].

Differences in findings between countries might be explained by the different contexts related to demographic and epidemiological changes [14, 31]. Moreover, there is heterogeneity concerning the methodology used, especially about the operationalization of multimorbidity and functional limitations. In addition, while the evidence has shown that the effects of multimorbidity on functional status may vary by age [33], the role of socioeconomic conditions in this context remains relatively unexplored [36, 37].

Thus, considering the demographic and epidemiologic modifications in the country [38] and the significant socioeconomic inequalities faced in Brazil [39], the objective of this study was to evaluate the association among functional limitations in basic and instrumental activities of daily living (outcomes), multimorbidity and socioeconomic position measures. Moreover, evaluated if the association between multimorbidity and the functional limitations (outcome) was different according to socioeconomic position measures.

## Materials and methods

### Study design and participants

This cross-sectional study used data from the older adults investigated by the National Health Survey (PNS), carried out in Brazil in 2019. The PNS is a population-based, household health survey carried out by the Ministry of Health in partnership with the Brazilian Institute of Geography and Statistics (IBGE), with a periodicity of approximately 5 years, being representative for the country, Federation Units, capitals, metropolitan regions and urban and rural areas.

The sampling was carried out using conglomerates in three stages of selection: 1) stratification of the primary sampling units which correspond to the Census Sectors or set of sectors; 2) selection by simple random sampling of a fixed number of permanent private households in each primary sampling unit. Households were selected from the National Register of Addresses for Statistical Purposes in its latest update; 3) selecting a resident of the household to answer the third part of the questionnaire [40].

This study included all individuals aged 60 or over who provided complete information for all the variables of interest.

This study used secondary data that is publicly available; therefore, it did not require approval from the Institutional Research Ethics Committee. The National Health Survey met all requirements for research involving human beings and was approved by the Brazilian National Research Ethics Committee/ National Health Council (protocol n° 3,529,376). Written informed consent was obtained for all the participants and the participant’s acceptance was registered directly on the mobile data collection devices before the interview [40].

### Variables

The outcomes of interest were functional limitations in basic (BADL) and instrumental activities of daily living (IADL). Six BADLs (feeding, bathing, using the toilet, dressing, crossing a room on the same floor and getting out of bed) and four IADLs (shopping, managing money, taking medication and using transportation) were assessed. Respondents who reported difficulty or inability to perform one or more of the tasks were recorded as having limitations in BADL or IADL [40].

The independent variables of interest were multimorbidity and socioeconomic measures. Multimorbidity was defined as the presence of two or more chronic diseases, as proposed by the World Health Organization [14]. All the diseases were self-reported and included the following conditions: high blood pressure, diabetes, high cholesterol, heart disease, stroke, asthma, arthritis or rheumatism, chronic back problem, chronic obstructive pulmonary disease, work-related musculoskeletal disorder, cancer, chronic kidney failure, depression, other mental illness. Each of the diseases was evaluated through the following question: “Has a doctor ever diagnosed you with [name of disease]?”. To assess the prevalence of depression and other mental diseases, the following questions were used, respectively: “Has a doctor or mental health professional (such as a psychiatrist or psychologist) ever given you a diagnosis of depression?” and “Has a doctor or healthcare professional (such as a psychiatrist or psychologist) ever given you the diagnosis of another mental illness?” For chronic spine pain "Do you have any chronic spine pain, such as chronic back or neck pain, lower back pain, sciatica, vertebrae or disc problems?".

The socioeconomic position measures were: 1) per capita household income (up to 1 minimum wage, >1–2 minimum wages, >2–3 minimum wages; >3 minimum wages); 2) education (no education/incomplete primary education; complete primary/incomplete secondary education; complete secondary education/ incomplete higher education; complete higher education); and 3) asset score in quintiles. The asset score was constructed using principal component analysis using information on household ownership of durable goods and housing characteristics based on the following information: household goods [internet, television, refrigerator, washing machine, computer, landline telephone, microwave, motorcycle, car] and household characteristics [number of rooms, presence of a maid, water supply source (deficient [shallow well, water table or well, fountain or spring, stored rainwater, other], adequate [general distribution network, deep or artesian well]), disposal of sewage from the bathroom (deficient [rudimentary septic tank, ditch, river, lake, stream or sea, other], adequate [general sewer or rainwater network, septic tank connected to the network, septic tank not connected to the network]).

The other covariates included were to adjust the models due to its association with the variables of interest: gender; age (60–69; 70–79; 80+); marital status [with or without a marital relationship (yes, no)]; and health-related behaviors [physically active (yes; no); smoking (yes, no)]. Those who performed vigorous exercise (running/ jogging, treadmill running, weight training, aerobics/spinning/step/jump, soccer, basketball and tennis) for 75 minutes or more per week, or light to moderate activities (walking, treadmill walking, aqua aerobics, general/localized gymnastics/pilates/stretching/yoga, swimming, martial arts/fighting, bicycle/electric bike, volleyball, dance and other activities) for 150 minutes or more per week were considered physically active [40].

### Statistical analysis

A descriptive analysis of the study variables was performed, followed by a bivariate analysis between the outcomes and all independent variables. The association between multimorbidity and dependent variables (IADL and BADL) was assessed using adjusted logistic regression models. First, the unadjusted association between each of the independent variables of interest and the outcomes were presented (Model 1). Second, a model with all the independent variables of interest adjusted for covariates was estimated and only the variables of interest that were significant were kept in the final model (Model 2). Finally, the moderating effect of socioeconomic conditions on the association between multimorbidity and functional limitations was assessed by including an interaction term between multimorbidity and the socioeconomic position measures that were kept in the final model (Models 3 and 4). Results were presented as odds ratios (OR) with 95% confidence intervals (95%CI). All analyses were performed using Stata 15.0.

## Results

The final sample consisted of 22,725 individuals, 56% were aged between 60 and 69 years, 56.7% were female, 58.3% had a marital relationship and 63.3% had up to incomplete primary education. The prevalence of functional limitation in BADL was 8.5% (95%CI: 7.9–9.2) and 18.6% (95%CI: 17.8–19.5) for IADL. Most of the older adults had multimorbidity [56.5% (95%CI: 55.4–57.6%)]. Of those with functional limitations in BADL, 20% were aged 80 years or more, 10.5% had no education or incomplete primary education, 10.7% had up to one minimum wage income and 11.3% had multimorbidity. Of those with IADL limitations, 6.9% were 80 years or more, 24% had no education or incomplete primary education, 24.4% had an income of up to one minimum wage and 23.9% had multimorbidity (Table 1).

**Table 1:** Descriptive analysis of study variables and the association between multimorbidity and independent variables.

	Total	BADL (yes)	IADL (yes)
	% (95%CI)	% (95%CI)	% (95%CI)
**Gender**			
Women	56.7 (55.6–57.7)	9.6 (8.7–10.6)	12.9 (12.1–13.6)
Men	43.3(42.3–44.4)	7.2 (6.4–8.0)	5.8 (5.3–6.3)
**Age (years)**			
60–69	56.3 (55.2–57.4)	5.6 (4.9–6.4)	5.1 (4.7–5.6)
70–79	30.1 (29.2–31.1)	8.9 (7.7–10.1)	6.6 (6.1–7.1)
80+	13.6 (12.8–14.3)	20 (17.7–22.5)	6.9 (6.4–7.5)
**Marital status**			
Single	41.7 (40.6–42.7)	10.8 (9.7–12.0)	10.7 (10.1–11.4)
Married/ partner	58.3 (57.3–59.4)	6.9 (6.2–7.7)	7.9 (7.3–8.5)
**Education** ^†^			
1	63.3 (62.1–64.5)	10.5 (9.6–11.4)	24 (22.8–25.2)
2	9.5 (8.9–10.2)	7.0 (5.4–9.0)	13.4(11.2–15.9)
3	15.9 (15.0–16.8)	4.8 (3.9–6.0)	9.9 (8.4–11.7)
4 (more)	11.3 (10.5–12.1)	4.1 (3.2–5.5)	5.3 (4.2–6.7)
**Income**			
Up to 1 MW	41.7 (40.6–42.9)	10.7 (9.6–11.8)	24.4 (23.1–25.9)
>1 MW up to 2 MW	31.9 (30.8–32.9)	8.0 (6.9–9.2)	17.1 (15.7–18.5)
>2 MW up to 3 MW	10.6 (10.1–11.5)	6.0 (4.6–7.9)	13.1 (11.1–15.5)
>3 MW	15.6 (14.7–16.6)	5.7 (4.6–7.0)	10.1 (8.6–11.8)
**Asset score**			
1^st^ quintile	20.1 (19.3–20.9)	10.3 (9.1–11.7)	26.3 (24.6–28.1)
2^nd^ quintile	20.1 (19.3–21.0)	10.7 (9.4–12.2)	23.4 (21.5–25.3)
3^rd^ quintile	20.5 (19.7–21.5)	8.7 (7.3–10.5)	17.8 (16.0–19.7)
4^th^ quintile	19.3 (18.4–20.3)	7.5 (6.0–9.3)	15.2 (13.4–17.3)
5^th^ quintile	19.9 (18.9–21.0)	5.3 (4.3–6.5)	10.3 (8.6–12.1)
**Smoking**			
Non-smoker	46.4 (45.2–47.5)	8.5 (7.6–9.5)	19.3 (18.0–20.6)
Current smoker	11.4 (10.8–12.1)	6.7 (5.3–8.5)	15 (12.9–17.3)
Former smoker	42.2 (41.1–43.3)	9.1 (8.1–10.1)	18.9 (17.6–20.3)
**Physical activity**			
No	80.2 (79.3–81.1)	10.0 (9.2–10.7)	21.8 (20.08–22.8)
Yes	19.8 (18.9–20.7)	2.8 (2.0–3.8)	6.0 (4.8–7.3)
**Multimorbidity**			
Yes	56.5(55.4–57.6)	11.3 (10.4–12.3)	23.9 (22.7–25.2)
No	43.3(42.4–44.6)	4.9 (4.3–5.6)	11.8 (10.8–12.9)

^†^Education: 1 = no education / incomplete primary education; 2 = complete primary/incomplete secondary education; 3 = secondary education complete/ uncompleted higher education; 4 = complete higher education or more. MW: minimum wage. BADL: basic activity of daily living. IADL: instrumental activity of daily living (IADL).

Table 2 shows the association between the functional limitation in BADL and the independent variables of interest. In the unadjusted analysis, it was found that older adults with multimorbidity were more likely to have BADL limitation [OR: 2.45 (95% CI: 2.07–2.89)] and for all socioeconomic variables, there was a negative relationship with BADL limitation (Model 1). In the final model, adjusted for covariates and for socioeconomic variables, individuals with multimorbidity had higher odds of BADL limitation [OR 2.30 (95%CI: 1.93–2.74)]. Regarding socioeconomic status, only education and income remained associated with the outcome. Individuals with complete secondary education/ incomplete higher education and complete higher education had 35% [OR: 0.65 (95%CI: 0.50–0.85)] and 36% [OR: 0.64 (95%CI: 0.44–0.94)] less odds of BADL limitation, respectively, than the ones with no education/incomplete primary education. Those with up to 2 MW; with up to 3; and those with more than 3 minimum wages had 22% [OR: 0.78 (95%CI: 0.63–0.95)], 33% [OR: 0.67 (95%CI: 0.47–0.95)] and 24% [OR:0.76 (95%CI: 0.58–1.00)] lower odds of BADL limitation, respectively, than older adults with up to 1 minimum wage. No interaction was observed between multimorbidity and the measures of socioeconomic position [income (p for interaction: 0.706) and education (p for interaction: 0.313)].

**Table 2:** Logistic regression models for the association between functional limitation in BADL, multimorbidity and socioeconomic conditions.

	BADL			
	Model 1^a^	Model 2^b^	Model 3^b^	Model 4^b^
	OR (95%CI)	OR (95%CI)	OR (95%CI)	OR (95%CI)
**Multimorbidity (yes)**	2.45 (2.07;2.89)***	2.30 (1.93; 2.74)***	2.29 (1.86; 2.82)***	2.35 (1.85; 2.99)***
**Asset score (ref**^†^ **1st quintile)**				
2^nd^ quintile	1.04 (0.85; 1.28)	-	-	-
3^rd^ quintile	0.83 (0.65;1.06)	-	-	-
4^th^ quintile	0.70 (0.53;0.92)*	-	-	-
5^th^ quintile	0.48 (0.37;0.63)***	-	-	-
**Education**^‡^ **(ref 1)**				
2	0.64 (0.48;0.86)**	0.88 (0.64; 1.21)	0.93 (0.58; 1.50)	0.88 (0.64; 1.21)
3	0.43 (0.34;0.55)***	0.65 (0.50; 0.85)***	0.49 (0.30; 0.80)**	0.65 (0.50; 0.84)**
4 (more)	0.36 (0.26;0.50)***	0.64 (0.44; 0.94)*	0.84 (0.48; 1.48)	0.64 (0.44; 0.94)*
**Income (Up to 1 MW** ^††^ **)**				
>1 MW up to 2 MW	0.73 (0.60;0.88)**	0.78 (0.63; 0.95)*	0.77 (0.63; 0.95)*	0.73 (0.53; 1.01)
>2 MW up to 3 MW	0.54 (0.40;0.73)***	0.67 (0.47; 0.95)*	0.67 (0.48; 0.95)*	0.86 (0.46; 1.61)
>3 MW	0.50 (0.40;0.64)***	0.76 (0.58; 1.00)*	0.76 (0.58; 1.00)*	0.83 (0.54; 1.27)
Multimorbidity*education				
Yes/education = 2			0.93 (0.53; 1.63)	
Yes/education = 3			1.45 (0.85; 2.47)	
Yes/education = 4			0.68 (0.34; 1.35)	
Multimorbidity*income				
Yes/>1 MW up to 2 MW				1.07 (0.72; 1.60)
Yes/ >2 MW up to 3 MW				0.70 (0.34; 1.46)
Yes/ >3 MW				0.89 (0.55; 1.44)

p-value:

*< 0.05,

**< 0.01,

***< 0.001.

^†^ref = reference category.

^‡^Education: 1 = no education / incomplete primary education; 2 = complete primary/incomplete secondary education; 3 = secondary education complete/ uncompleted higher education; 4 = complete higher education or more.

^††^MW: minimum wage.

^a^Unadjusted models.

^b^Model adjusted for age, sex, physical activity and smoking.

The results for the association between functional limitation in IADLs and the independent variables are presented in Table 3. In the unadjusted analysis (Model 1), it was found that older adults with multimorbidity were more likely to have functional limitations in IADLs [OR: 2.35 (95%CI: 2.08–2.64)]. Functional limitation in IADL was also associated with all socioeconomic variables. In the adjusted multiple model (Model 2), functional limitation in IADL was associated with multimorbidity and two of the socioeconomic position measures; education and income variables. Older adults with higher education were less likely to have functional limitations in IADLs. Individuals with up to 2 minimum wages; with up to 3; and those with more than 3 minimum wages had 34% [OR: 0.66 (95%CI: 0.57–0.75)], 43% [OR: 0.57 (95%CI: 0.45–0.72)] and 45% [OR: 0.55 (95%CI: 0.43–0.70)] less odds of IADL limitation, respectively than those with up to 1 minimum wage. There was no interaction between multimorbidity and socioeconomic variables [education (p for interaction: 0.832); income (p for interaction: 0.989).

**Table 3:** Logistic regression models for association between functional limitation in IADLs, multimorbidity and socioeconomic conditions.

	IADL			
	Model 1^a^	Model 2^b^	Model 3^b^	Model 4^b^
	OR (95%CI)	OR (95%CI)	OR (95%CI)	OR (95%CI)
**Multimorbidity (yes)**	2.35 (2.08;2.64)***	2.26 (1.98; 2.57)***	2.20 (1.91; 2.53)***	2.26 (1.91; 2.68)***
**Asset score (ref**^†^ **1st quintile)**				
2^nd^ quintile	0.85 (0.74;0.98)*	-	-	-
3^rd^ quintile	0.61 (0.52;0.71)***	-	-	-
4^th^ quintile	0.50 (0.42;0.60)***	-	-	-
5^th^ quintile	0.32 (0.26;0.40)***	-	-	-
**Education**^‡^ **(ref 1)**				
2	0.49 (0.40;0.60)***	0.75 (0.59;0.96)*	0.74 (0.52; 1.06)	
3	0.35 (0.29;0.43)***	0.61 (0.50;0.75)***	0.51 (0.32; 0.80)**	
4 (more)	0.18 (0.14;0.23)***	0.38 (0.27;0.53)***	0.38 (0.23; 0.63)***	
**Income (Up to 1 MW** ^††^ **)**				
>1 MW up to 2 MW	0.62 (0.55;0.70)***	0.66 (0.57;0.75)***	0.66 (0.57; 0.75)***	0.67 (0.53; 0.84)**
>2 MW up to 3 MW	0.47 (0.38;0.57)***	0.57 (0.45;0.72)***	0.58 (0.46; 0.72)***	0.55 (0.35; 0.85)**
>3 MW	0.35 (0.29;0.42)***	0.55 (0.43;0.70)***	0.55 (0.43; 0.70)***	0.55 (0.34; 0.91)*
Multimorbidity*education				
Yes/education = 2			1.03 (0.66; 1.59)	0.75 (0.59; 0.96)*
Yes/education = 3			1.29 (0.75; 2.23)	0.61 (0.50; 0.75)***
Yes/education = 4			0.99 (0.55; 1.77)	0.38 (0.27; 0.53)***
Multimorbidity*income				
Yes/>1 MW up to 2 MW				0.97 (0.74; 1.29)
Yes/ >2 MW up to 3 MW				1.07 (0.64; 1.78)
Yes/ >3 MW				0.99 (0.58; 1.70)

p-value:

*< 0.05,

**< 0.01,

***< 0.001.

^†^ref = reference category.

^‡^Education: 1 = no education / incomplete primary education; 2 = complete primary/incomplete secondary education; 3 = secondary education complete/ uncompleted higher education; 4 = complete higher education or more.

^††^MW: minimum wage.

^a^Unadjusted models.

^b^Model adjusted for age, sex, physical activity and smoking.

**Fig 1:**
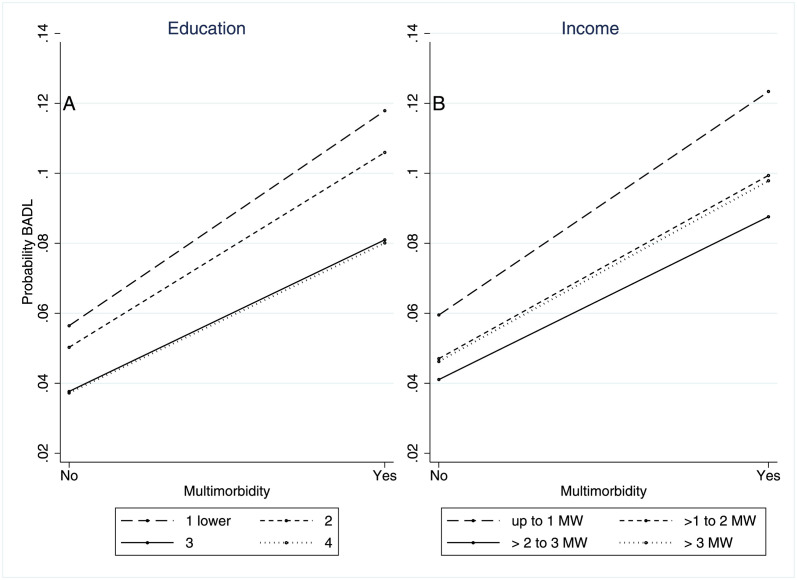
Probability of functional limitation in ABVD by multimorbidity and measures of socioeconomic position measures. Note: Education: 1 = no education/incomplete primary education; 2 = complete primary/incomplete secondary education; 3 = secondary education complete/ uncompleted higher education; 4 = complete higher education or more.

**Fig 2:**
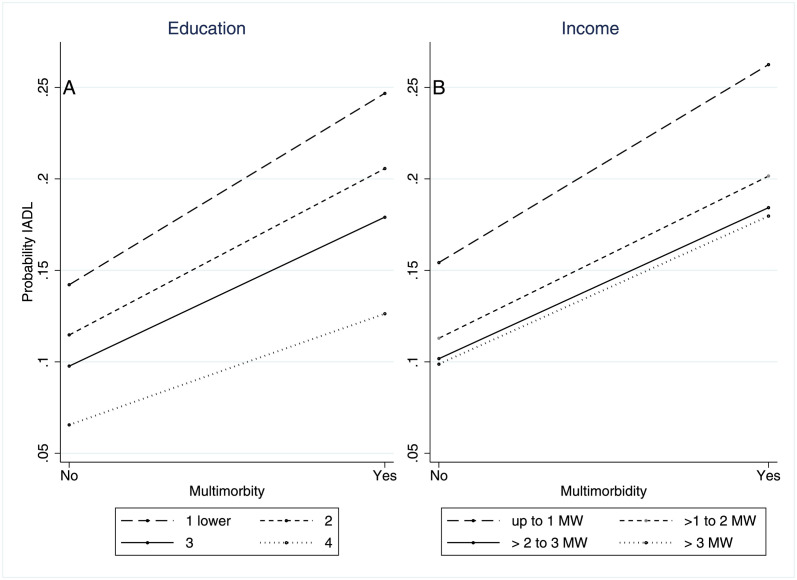
Probability of functional limitation in IADL by multimorbidity and measures of socioeconomic position measures. Note: Education: 1 = no education/incomplete primary education; 2 = complete primary/incomplete secondary education; 3 = secondary education complete/ uncompleted higher education; 4 = complete higher education or more.

Figs 1 and 2 show the probability of functional limitation in BADL and IADL, respectively, according to the presence of multimorbidity and levels of education and income. Older adults with multimorbidity have higher probabilities of limitation in BADL and IADL. The probabilities are higher among individuals with lower levels of education and income. However, the effect of multimorbidity is constant across different levels of socioeconomic status.

## Discussion

This study found that both outcomes, limitations in BADL and IADL, are associated with multimorbidity and different socioeconomic position measures. However, the effect of multimorbidity on either BADL or IADL was similar for different levels of socioeconomic position measures.

The positive association between multimorbidity and functional limitations in BADL and IADL confirms the results observed in longitudinal [33, 41] and cross-sectional studies [32, 34]. However, different methods for assessing activities of daily living [e.g. different scales and instruments] and multimorbidity [e.g. number and type of chronic diseases] may explain the differences in the magnitude of effects found in the previous studies.

In the present study, limitations in BADL and IADL were assessed independently and classified according to the presence of great difficulty or inability to perform the activities. Among the longitudinal studies, some assessed only BADL [41, 42], others activities of daily living together [43] and some assessed both BADL and IADL separately and considered as functional limitations the report of any difficulty in performing the activities [33]. Most longitudinal studies have shown that increasing numbers of diseases [33] and combinations of specific diseases [42] significantly increased the risk of functional decline [44, 45].

A longitudinal study that used data from the China Health and Retirement Longitudinal Study (CHARLS) and the Survey of Health, Ageing and Retirement in Europe (SHARE) found that the presence of multimorbidity and the increase in the number of chronic diseases were significantly associated with the incidence of functional limitations in BADL and IADL. In both study samples, those with one, two, three, four or more diseases were more likely to have functional limitations than the ones without chronic diseases, and this association increased concomitantly with the increase in the number of chronic diseases [33]. Sheridan et al. [46] found that multimorbidity combined with depressive symptoms was associated with higher odds of functional limitations in BADL and IADL. On the other hand, an investigation conducted in Norway that evaluated the association of complex multimorbidity (3 or more chronic diseases) with functional limitations in BADL and IADL did not find the association for basic activities of daily living [35].

In Brazil, the direct association between multimorbidity and functional limitation has not been fully explored. One cross-sectional study with data from the National Health Survey, conducted in 2013, found a positive association between three patterns of multimorbidity (cardiopulmonary pattern, vascular-metabolic pattern and the mental-musculoskeletal pattern) and functional limitations in BADL and IADL [32]. Among the studies that analyze the same patterns of multimorbidity, a study conducted in Mexico had similar results, with positive associations between the patterns and the presence of functional disability [47].

The association between socioeconomic conditions and functional limitations has been consistently reported [23, 48]. Previous findings have shown that individuals with lower education [37], income [15] or living in areas of lower socioeconomic status [17] had a higher prevalence and incidence of functional limitation. Confirming these findings, the present study found that individuals with higher income and education levels had lower odds of functional limitations in BADL and IADL. However, caution is needed when comparing results from different socioeconomic contexts and different stages of the demographic and epidemiologic transitions [38]. In this spectrum, it urges us to consider the significant socioeconomic inequalities faced in Brazil, which have extensively influenced health outcomes [39]. Our results showed that education and income, but not asset score, remained associated with BADL and IADL after adjustment for covariates. This result may reflect the relationship between income and education with the availability of material resources for access to health services [49].

Socioeconomic polices are well recognized to produce better health indicators, mainly those related to education investments [5]. The level of education itself can contribute to the improvement of the individual ability to interpret and understand health-related information. Furthermore, higher schooling may provide access to the best jobs as well as social goods and services, including health ones [50]. In the same way, investments in health systems can contribute to attenuating health inequalities related to socioeconomic conditions since these policies are proposed to promote equity reaching the most vulnerable groups as older adults and those in lower socioeconomic conditions [49].

Regarding the moderating effect of socioeconomic conditions on the relationship between multimorbidity and functional limitations, few studies had explored this issue with heterogeneous findings. Among previous studies [29, 36, 37], only a cross-sectional study [37] found a significant interaction. The present study found no distinct effect of multimorbidity on functional limitations at different levels of socioeconomic status.

The absence of this effect could be explained by the fact that socioeconomic conditions may act as a more distal determinant, preceding both multimorbidity and functional limitation. Education, for instance, could precede the occurrence of health problems, as it is determined in early adulthood [51], and income is influenced by the level of education [52]. When there is both low income and low educational attainment, their influence on health outcomes, specifically functional limitation, may overlap [53]. Singh-Manoux et al. [54] found that, over the age of 50, while clinical risk factors were predictors of the incidence of cardiometabolic disease, socioeconomic and behavioral factors of middle age were stronger predictors of the progression of cardiometabolic disease to multimorbidity and mortality, reinforcing the importance of socioeconomic conditions in the occurrence of multimorbidity.

While these findings are confirmatory, they hold significant public interest, especially in light of the growing population of older adults living with multiple chronic conditions and the increasing prevalence of individuals facing challenging socioeconomic conditions. Consequently, it becomes imperative to focus on preventing chronic diseases and ensuring universal access to multidisciplinary healthcare for those with multimorbidity. Although our findings align with previous research, which indicates a higher likelihood of functional limitations among those in lower socioeconomic positions, it is essential to emphasize that these observations should not merely be seen as confirmatory as health inequalities should and can be avoided. The necessity of monitoring health inequalities should be embraced as a fundamental public health policy. Therefore, the socioeconomic inequalities related to the functional limitations observed in the Brazilian older adult population underscore the pressing concern that our healthcare system is falling short in meeting the diverse healthcare needs of the population.

Among the strengths of this study, the use of data from the National Health Survey, a representative sample of the Brazilian population aged 60 years and over might be acknowledged. Another strong point was the assessment of functional limitations in BADL and IADL individually as there is a hierarchical relationship between them [35]. Finally, this study also advanced by investigating whether the association between functional limitation and multimorbidity could be moderated by different measures of socioeconomic status, specifically income, education and asset score. Regarding the limitations, the cross-sectional design of this study does not allow the establishment of a causal relationship between functional limitation, multimorbidity and socioeconomic conditions. Another possible limitation is the assessment of chronic diseases through self-report. However, it is a validated method in epidemiological studies and the most common data source in studies that assess multimorbidity [55].

In conclusion, this study found that multimorbidity is associated with functional limitation in basic and instrumental activities of daily living and this association is similar between different levels of education and income. Understanding the links between functional limitation, multimorbidity and socioeconomic conditions can help to identify the most vulnerable populations that need more health care assistance. Addressing multimorbidity and functional limitations from a public health perspective contributes to the promotion of self-care management strategies and reinforces the importance of longitudinal care. Thus, future research should longitudinally evaluate these associations and keep monitoring health inequalities on these outcomes.
